# Klotho: A Major Shareholder in Vascular Aging Enterprises

**DOI:** 10.3390/ijms20184637

**Published:** 2019-09-19

**Authors:** Kenneth Lim, Arvin Halim, Tzong-shi Lu, Alan Ashworth, Irene Chong

**Affiliations:** 1 Division of Nephrology, Department of Medicine, Massachusetts General Hospital and Harvard Medical School, Boston, MA 02114, USA; 2 MGH Renal Associates, 165 Cambridge Street, Suite 302, Boston, MA 02114, USA; 3 Renal Division, Department of Medicine, Brigham and Women’s Hospital and Harvard Medical School, Boston, MA 02114, USA; arv.halim@gmail.com (A.H.);; 4 Helen Diller Family Comprehensive Cancer Center, University of California San Francisco (UCSF), 1450 3rd St, San Francisco, CA 94158, USA; Alan.Ashworth@ucsf.edu; 5 The Institute of Cancer Research and The Royal Marsden NHS Foundation Trust, Chester Beatty Laboratories, 237 Fulham Road, London SW3 6JB, UK; Irene.Chong@icr.ac.uk

**Keywords:** vascular aging, vascular calcification, arteriosclerosis, Klotho, chronic kidney disease (CKD), cancer, diabetes

## Abstract

Accelerated vascular aging is a condition that occurs as a complication of several highly prevalent inflammatory conditions such as chronic kidney disease, cancer, HIV infection and diabetes. Age-associated vascular alterations underlie a continuum of expression toward clinically overt cardiovascular disease. This has contributed to the striking epidemiologic transition whereby such noncommunicable diseases have taken center stage as modern-day global epidemics and public health problems. The identification of α-Klotho, a remarkable protein that confers powerful anti-aging properties has stimulated significant interest. In fact, emerging data have provided fundamental rationale for Klotho-based therapeutic intervention for vascular diseases and multiple other potential indications. However, the application of such discoveries in Klotho research remains fragmented due to significant gaps in our molecular understanding of Klotho biology, as well as hurdles in clinical research and experimental barriers that must first be overcome. These advances will be critical to establish the scientific platform from which future Klotho-based interventional trials and therapeutic enterprises can be successfully launched.

## 1. Introduction

Advancing age is a major risk factor for both subclinical and clinically overt cardiovascular disease (CVD) [1]. Age-associated changes of cardiovascular structure and function occur universally in apparently healthy persons without overt clinical consequences. However, these changes can compromise cardiovascular reserve capacity, alter the threshold for symptoms and signs, and can occur at an accelerated rate in various disease states thereby leading to occult CVD [1]. Features of accelerated or premature cardiovascular aging and reduced lifespan accompany patients with a number of chronic disease states, including chronic kidney disease (CKD), cancer, diabetes, HIV infection and inflammatory arthropathies [2,3]. It is becoming increasingly apparent that premature age-related vascular alterations compound in different vascular beds to cause an exponential increase in disability and function as a major contributor to occult CVD [4]. Models that include population aging, increasing rates of urbanization and globalization and increasing prevalence of these chronic disease states have demonstrated a striking epidemiologic transition whereby noncommunicable diseases such as accelerated CVD, have taken over as a modern-day global public health problem and a leading cause of death [5].

The kidneys are among the organs that are functionally most sensitive to the aging process, and the link between aging and kidney function is well-recognized and appears bidirectional [2,6]. Premature CVD is well-illustrated in CKD patients by the observation that cardiovascular mortality in a 20 year-old dialysis patient is similar to that of an 80 year-old person without significant renal impairment [7]. In fact, CVD remains the leading cause of death in patients with CKD [7,8,9]. The uremic phenotype in CKD recapitulates many features of aging, such as arteriosclerosis, atherosclerosis, osteoporosis, poor wound healing, sarcopenia, inflammatory and oxidative stress, insulin resistance, frailty, hypogonadism, skin atrophy, cognitive dysfunction and disability; therefore, CKD has been increasingly recognized as a model for premature aging syndrome [2]. Age-associated vascular changes in CKD is a complex process driven by both traditional and CKD-related risk factors, such as uremia, mineral disorders, fibroblast growth factor (FGF)-23, inflammation, post-translational protein modifications, metabolites, advanced glycation end products and pressure and volume overload [10,11,12,13]. Similarly, inflammation and oxidative stress beyond traditional risk factors are also key contributors to premature vascular aging in other conditions, such as in cancer and diabetes [14,15]. In survivors of cancer, the direct effects of various chemotherapies and radiation on telomere length, senescent cells, epigenetic modifications and microRNAs have also been linked to accelerated aging [16].

Aging and age-associated changes of the vascular system involve fundamentally different alterations from atherosclerosis. Vascular alterations attributable to aging include arterial dilatation, vascular calcification, increased collagen-to-elastin ratio with fragmentation, endothelial dysfunction and hypertrophy of vascular smooth muscles cells (VSMCs) [17]. These changes are typically described as arteriosclerosis and provide the milieu for the development of overt vascular disease; this interaction between age-associated changes and the development of clinically overt disease has previously been termed the “vascular aging-vascular disease” pathway [18,19,20]. The terms “arteriosclerosis” and “atherosclerosis” have long been frequently confused, however they represent distinct groups of pathologic processes: atherosclerotic disease primarily affects the intima leading to plaque formation, while arteriosclerotic disease is primarily a disease of the medial layer of arteries.

Medial vascular calcification occurs at an accelerated rate in age-associated conditions such as CKD, cancer and diabetes with the consequence of loss of arterial distensibility and increasing arterial stiffening. As arteries stiffen, this leads to a lack of buffering capabilities during oscillatory changes in blood pressure caused by intermittent ventricular ejection and results in a more rigid aorta that can accommodate less stroke volume; these changes can be clinically detected by greater pressure augmentation in systole and higher pulse pressures [17]. End organs such as the heart, brain and kidneys are consequently exposed to higher systolic pressures and greater pressure fluctuations resulting in microvascular damage, reduced diastolic coronary perfusion and promotion of subendocardial ischemia and myocardial fibrosis. Additionally, age-associated arterial hardening leads to left ventricular hypertrophy (LVH), increased cardiac afterload and characteristic diastolic failure [18,19,20].

α-Klotho, hereby referred to as Klotho, was first discovered by Kuro-o et al. in 1997 and has made a triumphant entry onto the center stage in the field of aging [21]. The identification of Klotho as a novel anti-aging protein has become a major focal point of aging research and challenged the long-held paradigm of aging as a passive, inevitable process of deteriorating systemic organ function. Klotho knockout mice demonstrate a premature aging syndrome phenotype and exhibit shortened life span [21]. Restoration of Klotho in Klotho-deficient mice however ameliorated these changes and these mice live 30% longer than wild-type [21,22,23]. The kidney is the primary organ of functional Klotho expression and its expression has been consistently linked with kidney function. Analysis of 2946 participants within the Healthy Aging and Body Composition Study demonstrated that a high soluble Klotho level was independently associated with a lower risk of decline in kidney function [24]. In fact, total Klotho protein levels decline in serum as kidney dysfunction ensues in CKD and with advancing age [25]. Critically, accumulating experimental, clinical and epidemiologic evidence have shown that Klotho deficiency is associated with a variety of vascular outcomes, such as cardiovascular events and arterial stiffness [26], vascular calcification [13,27] and atherosclerosis [28]. Moreover, therapeutic restoration of Klotho can ameliorate these changes [21,22,23,27].

Taken together, emerging data suggest that arterial aging and age-associated arterial diseases are active, tightly regulated cell-mediated processes that may be potentially modifiable. Given the implications of these discoveries for human health, Klotho as a potential longevity-modulating therapeutic strategy has attracted widespread attention and as a result, the field of aging research has flourished over the past decade. This review will consider the biology of Klotho and contemporary evidence for its therapeutic applications in the treatment of age-associated vascular diseases [21,29].

## 2. The Biology of Klotho

A significant advance in the field of aging research came about in 1997 after the serendipitous discovery of the aging-suppressor gene named *Klotho* [21]. The gene was named after Clotho, a figure in ancient Greek mythology responsible for spinning the thread of human life. Mice lacking the *Klotho* gene develop an aging-like phenotype similar to premature human aging, including endothelial dysfunction, vascular calcification, progressive atherosclerosis and myocardial hypertrophy of the cardiovascular system [21,27,30]. These mice develop aging-like changes beginning at three weeks of age at which time their growth is stunted and have an average life span of 15 weeks, compared with wild-type mice that have an average lifespan of approximately 2.5–3 years.

### 2.1. Structure of Klotho

The *Klotho* gene spans approximately 50 kb and is composed of five exons located on chromosome 13q12 in humans [29]. *Klotho* encodes a type 1 transmembrane glycoprotein that is 1014 and 1012 amino acids long in mouse and human, respectively [21,29]. Full-length Klotho consists of a putative N-terminal signal sequence and two internal repeats (mKL1 and mKL2) constituting the extracellular domain, a single-pass membrane spanning domain, and a short C-terminal intracellular domain of 10 amino acids [29]. Full-length Klotho is an approximately 135 kDa in molecular weight, the size being influenced by N-glycosylation [31]. Klotho also exists in the circulation as both soluble and secreted isoforms [31]: Soluble Klotho is generated from cleavage of full-length Klotho by membrane proteases (ADAM10 and ADAM17) in an α-cut to generate a 130 kDa protein that contains both the KL1 and KL2 domains, but lacks the transmembrane and intracellular components. Following the α-cut, the remaining transmembrane and cytosolic 5 kDa portion then undergoes proteolysis by γ-secretase [32,33]. A 65 kDa isoform is also generated by a β-cut and contains the KL1 domain.

In the mouse an alternatively spliced *Klotho* mRNA is produced which lacks exons 4 and 5, and in humans a premature stop codon leads to the truncation of the Klotho protein [29,34]. Therefore, a 65 kDa fragment consisting of the KL1 domain was also previously thought to be secreted by alternative splicing in humans [31]. However, this paradigm was challenged in a recent study by Mencke et al. that reported that the premature stop codon responsible for the truncated form primes the alternatively spliced mRNA for degradation [35]. Therefore, these data now suggest that the secreted Klotho isoform does not exist in humans. It remains unclear whether a secreted Klotho isoform exists in mice.

The KL1 and KL2 domains have sequence similarity to family 1 β-glycosidases and are most similar to mammalian lactase–phlorizin hydrolase (LPH) [21]. Although the internal repeats lack the prototypical catalytic glutamic acid residues of β-glycosidases, they have substitute catalytic acid-base Asn in KL1 and a nucleophilic Ser residue in KL2 for humans. Fluorescence assays with chimeric 958 amino acid-long mouse Klotho extracellular domain with human immunoglobulin Fc suggested the internal repeats have β-glucouronidase activity [36]. However, in a recent landmark study by Chen et al. using 3.0 Å resolution crystallography of a 1:1:1 ternary complex of soluble Klotho bound to FGF23 and fibroblast growth factor receptor (FGFR)1c, refuted the hypothesis that Klotho has intrinsic catalytic activity. They confirmed the absence of catalytic Glu residues in the putative catalytic pockets of KL1 and KL2, but also showed that soluble Klotho did not hydrolyze the substrates of β-glucouronidase and sialidase, in vitro [37]. With crystallography evidence showing that KL1 and KL2 each appear to take on a (βα)_8_ riosephosphate isomerase (TIM) barrel fold, Chen et al. suggested that Klotho is the first and so-far only known TIM barrel protein that acts only as a non-enzymatic molecular scaffold [37].

### 2.2. Tissue Expression of Klotho

Studies in the mouse originally identified Klotho expression mainly in distal convoluted tubule cells and to a lesser extent, in the proximal convoluted tubule cells of the kidneys [21,38]. In an elegant study by Lindberg et al. a novel mouse strain with the *Klotho* gene deleted throughout the nephron was generated and this was found to exhibit an 80% reduction in circulating Klotho levels confirming that the kidneys are the primary source of soluble Klotho [39]. These findings are consistent with observations in human patients who show an approximately 30% reduction in circulating Klotho following unilateral nephrectomy [40].

In a study by Lim et al., that characterized systemwide tissue expression of transmembrane Klotho using targeted proteomic analysis in parallel with conventional antibody-based methods, Klotho expression was identified in a variety of organ systems, including arterial (in both endothelial cells and vascular smooth muscle cells (VSMCs)), epithelial, endocrine, reproductive and neuronal tissues [3]. Extra-renal expression of Klotho appears to be less abundant than expression at the kidneys, and this suggests that local expression of Klotho could serve in an autocrine/paracrine fashion to regulate tissue health locally while the kidneys remain the principal source of endocrine Klotho. This situation mirrors the vitamin D hormonal system, whereby endocrine vitamin D is principally sourced from the kidney [41].

### 2.3. The Klotho–FGF23 Axis

A major but not exclusive role for Klotho is serving as an obligate coreceptor for fibroblast growth factor 23 (FGF23) signaling. FGF23 is a phosphaturic hormone that is secreted by osteocytes and plays a pivotal role in regulating phosphate homeostasis [42,43]. FGF23 knockout mice were found to develop a complex aging-like phenotype very similar to that observed in Klotho-deficient mice [44]. Both Klotho and FGF23 deficient mice develop hyperphosphatemia and high serum levels of active 1,25-dihydroxyvitamin D levels together with premature aging features [44,45]. However, most of these symptoms of premature aging are alleviated by feeding these mice a low phosphate diet to restore phosphate homeostasis, despite this diet stimulating further increases in vitamin D levels [46]. These results suggested that phosphate imbalance, rather than increased serum vitamin D levels, is a major regulator of aging as discussed below.

Klotho forms a complex with fibroblast growth factor receptors (FGFR) 1c, 3c or 4 which converts their canonical functions into specific receptors for FGF23 [47,48,49]. Both membrane-bound and soluble Klotho can function as a co-receptor for FGF23. Jimbo et al. showed that FGF23 enhanced extracellular signal-regulated kinases (ERK)1/2 phosphorylation in Klotho-overexpressing, but not in naive VSMCs without detectable Klotho [50]. Structural studies suggest soluble Klotho acts to enhance the binding affinity of FGF23 to FGFR1c via proximity, with the assistance of Zn^2+^ prosthetic group [37]. This is speculated to promote heparan sulfate (HS)-induced dimerization of FGF23–FGFR1c, which is likely required for FGF23-mediated FGFR1c activation [37]. Binding of FGF23 to the Klotho–FGFR complex induces the internalization and degradation of sodium-dependent phosphate transport protein 2A (NPT2A) and downregulates expression of *NPT2A* (SLC34AS1) at the local brush border membrane of proximal tubular cells [51]. Additionally, FGF23 suppresses the expression of Cyp27b1 which encodes 1α-hydroxylase, the enzyme that converts 25-hydroxyvitamin D_3_ to active 1,25-dihydroxyvitamin D_3_ and stimulates its metabolic breakdown by increasing expression of Cyp24a1 which encodes 24-hydroxylase [52].

These effects collectively operate to help maintain phosphate balance with significant implications on vascular health, given that phosphate is a major determinant of CVD in CKD and the aging process. High phosphate concentrations have been widely shown to stimulate endothelial and vascular smooth muscle cell (VSMC) damage and calcification [53,54]. Phosphate toxicity can result in the formation of calcium phosphate (CaPi) product; in the blood, CaPi binds to serum protein fetuin-A and forms colloidal particles termed calciprotein particles (CPP). CPP can induce endothelial damage, VSMC calcification and innate immune responses, thereby contributing to accelerated vascular aging [55]. Notably, phosphate retention, progressive hyperphosphatemia, rising FGF23 levels and low Klotho expression collectively are observed in human patients with advancing CKD and has been associated with progressive age-associated cardiovascular alterations [13,41]. This has prompted the view that CKD is a strategic clinical model to study premature cardiovascular aging [2]. These observations have consistently linked phosphate to the aging process and emphasize the importance of Klotho–FGF23 as a counterregulatory hormonal system. Of note, Klotho–FGF23 also regulates other channels in the kidney, such as members of the transient receptor potential (TRP) vanilloid (V) subgroup, TRPV5 and TRPV6 calcium channels and renal outer medullary potassium channel 1 [56]; these effects are beyond the scope of this review.

### 2.4. Klotho Exerts Pleiotropic Functions Independent of FGF23

In the absence of FGF23, soluble Klotho can directly exert phosphaturic activity by promoting the endocytosis and degradation of NPT2A, NPT3 and phosphate transporter 1 (PiT1) and 2 (PiT2) [57,58]. However, the role of soluble Klotho in regulating mineral metabolism has been subject to considerable controversy. Chen et al. showed that in vitro soluble and transmembrane Klotho possess similar capacities to facilitate FGF23 signaling [37]. Recombinant soluble Klotho injected into wild-type mice results in a small, but significant increase in urinary phosphate excretion. However, injection of a mutated soluble Klotho isoform lacking the FGF receptor binding arm resulted in a striking downregulation of FGF23 target genes in the kidney with the development of hyperphosphatemia. These findings suggest that the effects of soluble Klotho on mineral metabolism are FGF23 dependent.

Klotho can modulate a number of evolutionary conserved intracellular signaling pathways involved in longevity, including insulin and insulin-like growth factor 1 [59], target of rapamycin [60], cyclic adenosine monophosphate [61], protein kinase C [62], transforming growth factor-β [63], p53/p21 [64] and Wnt signaling [65]. Unfortunately, the mechanisms by which Klotho exerts anti-aging effects and how Klotho overexpressing mice live 20–30% longer compared to wildtype animals is still not well-understood.

## 3. Vasculo-Protective Effects of Klotho

Accumulating epidemiological and observational studies have linked circulating Klotho levels to cardiovascular risk and outcomes: In a study by Semba et al. that examined 804 community-dwelling adults aged 65 or greater, the investigators found that participants in the lowest tertile of plasma Klotho (<575 pg/mL) had an increased risk of death compared with participants in the highest tertile (>763 pg/mL; hazard ratio 1.78, 95% CI 1.20–2.63) [66]. Arking et al. examined the association of a functional variant of Klotho, termed KL-VS genotyped in 525 Ashkenazi Jews composed of 216 probands (age ≥ 95 years) and 309 unrelated individuals (ages 51 to 94) [67]. KL-VS was associated with cardiovascular disease risk factors including high-density lipoprotein (*p* < 0.05) and systolic blood pressure (*p* < 0.008). Furthermore, homozygous KL-VS individuals had the highest risk of vascular events (odds ratio (OR) 30.65; 95% CI 2.55–368) and a 4.49-fold (95% CI 1.35 to 14.97) relative risk for mortality. Similarly, several other studies have shown that plasma Klotho is inversely associated with CVD [66,68], and macrovascular disease (including coronary artery disease and cerebrovascular accidents) in type 2 diabetics [28,66].

**Figure 1:**
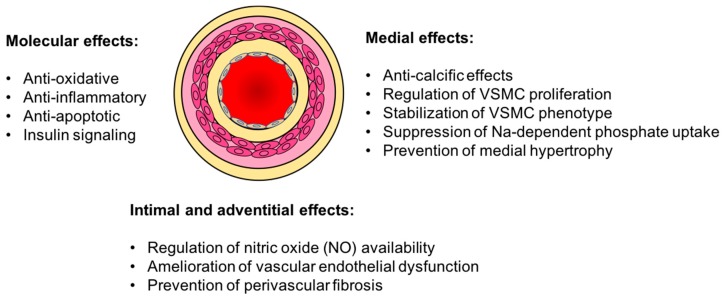
Vasculo-protective effects of Klotho. The presence of Klotho can exert pleiotropic protective effects against age-associated arterial changes. VSMC, Vascular Smooth Muscle Cells.

Mounting experimental evidence suggest that the presence of Klotho is critical for vascular health and its administration can exert vasculo-protective effects (Figure 1). Klotho knock-out mice develop striking vascular disease, including widespread vascular calcification, endothelial dysfunction and progressive atherosclerosis together with severe hypervitaminosis D, hypercalcemia and hyperphosophatemia [69]. Conversely, a growing body of evidence suggest that restoration of Klotho ameliorates these changes [21,22,23,27].

### 3.1. Vascular Calcification

Molecularly, age-associated changes of the arterial wall are characterized by osteogenic transformation of vascular smooth muscle cells (VSMCs) and loss of their contractile phenotype, upregulation of transcriptional regulators of osteoblastic differentiation such as Runx2, increased expression of bone markers (alkaline phosphatase, osteopontin, osteocalcin), release of matrix vesicles, apoptosis, extracellular matrix degradation and nuclear changes [70,71,72]. The continuum of expression of these molecular and cellular alterations underlie the development of vascular calcification, arteriosclerosis and arterial remodeling. Both the extent of vascular calcification and arterial stiffening is a hallmark of vascular aging and has been used as a measure of biological cardiovascular age [73,74,75]. Accumulation of biomarkers such as senescence associated β-galactosidase (SAβG) is also widely used to assess biological aging of the arterial tree [76].

Emerging data suggest that dysregulation of Klotho is centrally involved in the development of calcification and has provided a case to support potential therapeutic interference by restoration or supplementation of Klotho. Both endogenous tissue Klotho and soluble Klotho have been shown to exert anti-calcific effects: Hu et al. showed that transgenic CKD mice that overexpress Klotho had attenuated development of vascular calcification together with better renal function and enhanced phosphaturia, compared to wild-type mice with CKD [27]. Conversely, Klotho deficient mice with CKD developed severe calcification and worse renal function. Soluble Klotho directly suppressed Na-dependent uptake of phosphate and mineralization induced by high phosphate as well as preserving differentiation in VSMCs, in vitro. Similarly, intraperitoneal administration of recombinant Klotho [77], increased soluble Klotho by vitamin D receptor agonists (VDRA) treatment [69], and Klotho gene delivery were all associated with reduced vascular calcification [23].

Lim et al. were the first investigators to describe endogenous Klotho expression in human arteries and VSMCs [13]. They showed that CKD is a state of vascular Klotho deficiency that can be promoted by inflammatory, uremic and metabolic stressors. Klotho knockdown in VSMCs abrogated FGF23 mediated intracellular signaling and promoted the development of accelerated VSMC calcification, in vitro. Furthermore, restoration of Klotho deficiency by vitamin D receptor activators conferred responsiveness of VSMCs to potential FGF23 anti-calcific effects. Fang et al. showed that mice with early CKD by mild renal ablation developed a reduction in vascular Klotho expression together with vascular osteoblastic transition, increased osteocytic secreted proteins and inhibition of skeletal modeling, characteristic of mineral bone disorder (MBD) [78]. Several other studies have confirmed vascular expression of Klotho in various animal models [79,80,81,82], while others have not detected [83,84] or have not found changes in its vascular expression in CKD (Table 1) [50].

Deficiency of Klotho in VSMCs results in loss of smooth muscle cell contractile phenotype; similarly transformation of VSMCs from a contractile to a secretory phenotype has been associated with vascular Klotho deficiency [13]. These results suggest that endogenous Klotho expression is present only in VSMCs with a contractile phenotype.

### 3.2. Endothelial Dysfunction

Endothelial dysfunction is an early event in the development of atherosclerosis and encompasses a constellation of maladaptive alterations with a variety of implications, such as dysregulation of local vascular tone via regulation of nitric oxide (NO) availability, redox balance and orchestration of acute and chronic inflammatory reactions within the arterial wall [85]. Studies have shown that soluble Klotho decreases H_2_O_2_- and etoposide- induced apoptosis in human umbilical vascular endothelial cells (HUVECs). These anti-apoptotic effects occurred through the caspase-3/caspase-9 and p53/p21 pathways [64]. Six et al. showed that treatment of HUVECs with Klotho partially reverts FGF23-induced vasoconstriction, induced relaxation of preconstricted aorta by phosphate exposure and enhanced endothelial NO production [86].

Saito et al. showed that Klotho heterozygous mice exhibited attenuated aortic and arteriolar vasodilatation, however parabiosis between wild-type heterozygous Klotho mice restored endothelial function in heterozygous Klotho mice [87]. Additionally, heterozygous Klotho mice exhibited reduced nitric oxide metabolites (NO-2 and NO-3) in urine compared to wild-type mice, suggesting a decrease in NO production. In a related study, using the Otsuka Long-Evans Tokushima Fatty (OLETF) rat, an animal model with multiple atherogenic risk factors, adenovirus-mediated Klotho gene delivery ameliorated vascular endothelial dysfunction, increased nitric oxide production, reduced elevated blood pressure and prevented medial hypertrophy and perivascular fibrosis [88].

### 3.3. Oxidative Stress and Inflammation

Several studies have provided evidence that Klotho can suppress oxidative stress and inflammation, central processes firmly established in the development of vascular dysfunction, calcification and atherosclerosis. Klotho deficiency increases endogenous reactive oxygen species (ROS) generation and accentuates oxidative stress [89]. Conversely, overexpression of Klotho can decrease H_2_O_2_ induced-apoptosis, superoxide anion generation as well as β-galactosidase activity, mitochondrial DNA fragmentation, lipid peroxidation and Bax protein expression [58,89,90]. FOXO3a is a transcription factor that functions as a negative regulator of mitochondrial ROS generation [91]. It upregulates the expression of manganese superoxide dismutase (MnSOD), an important enzyme involved in mitochondrial antioxidant defense [22,59]. Klotho increases FOXO3a phosphorylation, suggesting that Klotho may suppress ROS-related oxidative stress. This is supported by observations that transgenic mice that overexpress Klotho have higher MnSOD expression and lower oxidative stress as evidenced by lower levels of urinary 8-hydroxy-2-deoxyguanosine, a marker of oxidative DNA damage [59,92,93]. Overexpression of Klotho or treatment with recombinant Klotho enhanced MnSOD expression, partially via activation of the cAMP signaling pathway [61]. In a study by Wang et al. the investigators found that Klotho gene transfer decreased nicotinamide adenine dinucleotide phosphate (NADPH) oxidase 2 (Nox2) protein expression, intracellular superoxide production and oxidative stress in rat aortic smooth muscle cells, in vitro [90]. Klotho gene expression also significantly attenuated angiotensin II (AngII)-induced superoxide production, oxidative damage and apoptosis. In another study, Klotho gene delivery in spontaneous hypertensive rats decreased upregulation of NADPH oxidase 2 activity and superoxide production and prevented the progression of spontaneous hypertension [94].

Anti-inflammatory actions of Klotho may underlie some of its vasculo-protective effects. Klotho protein has been shown to suppress the expression of intracellular adhesion molecule-1 (ICAM-1) and vascular cell adhesion molecule-1 (VCAM-1) in HUVECs exposed to tumour necrosis factor (TNF)-alpha [95]. These effects were associated with attenuation of nuclear factor (NF)-kappaB activation, IkappaB phosphorylation and inhibition of TNF-alpha induced monocyte adhesion. Liu et al. showed that intracellular, but not secreted Klotho interacts with retinoic-acid-inducible gene-I (RIG-I) thereby inhibiting RIG-I induced expression of interleukin (IL)-6 and IL-8 in senescent cells [96]. Using a senescence-accelerated mice P1 (SAMP1) aging model that developed aortic valve fibrosis, Chen et al. showed that adenovirus delivery of secreted Klotho inhibited inflammatory processes in aortic valves, including inhibition of monocyte chemoattractant protein-1 (MCP1), intercellualar adhesion molecule 1 (ICAM-1) expression, transforming growth factor (TGF)β upregulation, attenuated upregulation of tartrate-resistant acid phosphatase (TRAP) and matrix metallopeptidase (MMP)-2 expression and suppressed myofibroblastic transition [97].

## 4. Experimental Challenges and the Future of Klotho-Based Therapies

**Figure 2:**
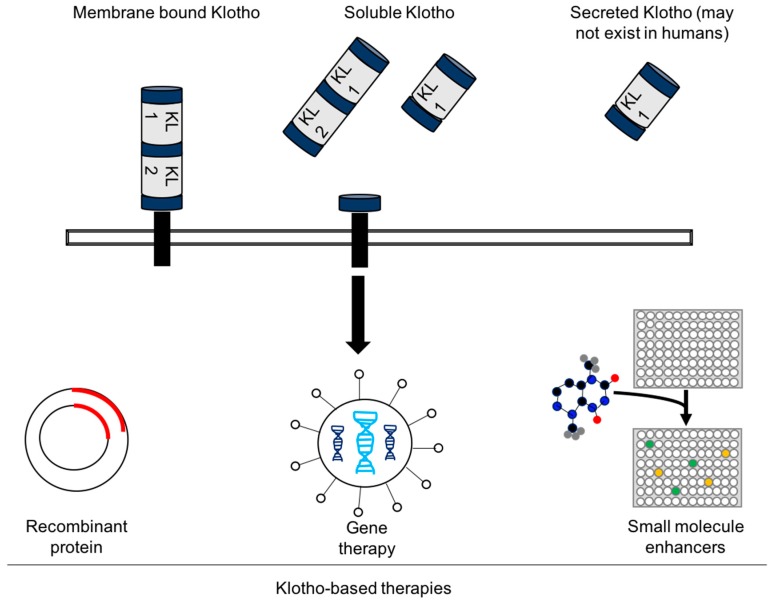
Potential delivery modalities of Klotho-based therapies. Full-length transmembrane Klotho is a ~135 kDa protein. Cleavage of full-length Klotho by membrane proteases (ADAM10 and ADAM17) in an α-cut generates a 130 kDa soluble isoform containing the KL1 and KL2 domains. Cleavage in a β-cut generates a 65 KDa isoform that contains only the KL1 domain. Recent evidence has challenged the existent of secreted Klotho by alternative splicing of Klotho mRNA. Various Klotho-based delivery strategies have been explored as illustrated.

Despite the growing number of promising basic science discoveries over the past two decades supporting the therapeutic potential of Klotho to modulate CVD and other disease phenotypes, there are currently no clinical trials exploring the efficacy of Klotho-based therapies. However, several different Klotho-based delivery strategies have been explored. These include recombinant Klotho protein, gene therapy delivery of Klotho or small molecules that can enhance Klotho expression (Figure 2) [98,99]. These various strategies are now employed in an emerging landscape of Klotho-based biotechnology start-up companies for various indications, such as “Klotho Therapeutics” (https://www.klotho.com/), “Klogene” (http://www.klogene.com/) and “Unity Biotechnology” (https://unitybiotechnology.com/).

Several critical considerations, gaps and challenges must first be overcome before Klotho-based interventions can be successfully translated into potential therapies for vascular diseases and beyond: Firstly, the precise functional role of the various Klotho isoforms in health and disease, their molecular mechanisms and the identity of the Klotho receptor for FGF23 independent effects remains to be elucidated. Therapeutic strategies will need to carefully target the isoform with most clinical benefit and carefully consider their application from a standpoint of prophylactic treatment (before overt clinical disease appears in high-risk patients) or reversal of existing vascular diseases. Given the pleiotropic effects of Klotho and its inherent large globular form, significant molecular analysis is still needed to identify the mechanisms and active sites of Klotho responsible for its various activities. This knowledge would form a critical platform on which successful pharmaceutical engineering can then be built.

From a clinical viewpoint, the precise concentrations of circulating Klotho and their specific isoforms that would be considered sufficient is still unknown and this is likely influenced by genetic variability between populations. Additionally, it is unknown whether supplementation above levels considered sufficient would be of therapeutic benefit or result in toxicity. In fact, high levels of Klotho may result in hypophosphatemic rickets and hyperparathyroidism [100]. These latter considerations are critical given that emerging concepts in precision medicine suggesting that individual responsiveness to therapeutic intervention is a function of naturally occurring genetic variants [101,102]. This would influence both the dosing and timing of Klotho delivery. Furthermore, studies are required to help guide the selection of patients who would qualify as good candidates for a Klotho-based intervention.

These experimental and clinical studies remain hampered by significant challenges with current antibody-based techniques for assessing Klotho: While commercially available antibodies against Klotho are available, most of them appear to be unspecific and cross-react with other proteins [103]. A novel synthetic anti-Klotho antibody (termed sb106) has been shown to detect Klotho in tissue and soluble Klotho in serum and urine, however this antibody is currently not commercially available [103,104]. Similarly, antibody-based assays for assessing serum Klotho levels have provided inconsistent results in CKD patients. In one study that compared a time-resolved fluorescence immunoassay (TRF, Cusabio, China) to an ELISA (IBL, Japan), surprisingly, no correlation was found between the assays and the levels of serum Klotho differed with by a factor 1000 [105]. At present, assessment of circulating Klotho using an immunoprecipitation-immunblot (IP-IB) assay has been shown to be highly correlated with glomerular filtration rate (GFR) in never-thawed serum samples of humans with varying severity of kidney disease compared to commercial ELISA [106]. Additionally, it is unclear whether commercial assays are detecting the 130 kDa and/or 65 kDa circulating isoforms.

A related technical challenge is that soluble Klotho appears to be highly unstable in blood and urine [104,107]. Prevention of degradation to conserve soluble Klotho, standardization of techniques and rigorous, in-house validation is therefore essential, however these have not been described. Similarly, generation of recombinant Klotho for experimental studies have been challenging and the variability and unpredictable quality of commercially available recombinant proteins may affect the reproducibility of reported effects [99]. Additionally, functional assays to detect Klotho activity are lacking [108]. Leveraging advances in next-generation sequencing technologies and Mendelian randomization studies are therefore imperative in the interim to first help identify genetic variants as instruments for strengthening causal inference in observation studies, while methodological improvements in antibody-based techniques and assays are being made.

## 5. Summary

The central thesis of this review was that accumulating evidence has stimulated significant interest and provided fundamental rationale for the therapeutic role of Klotho for age-related vascular diseases. As such, there is much reason for optimism toward the development of Klotho-based therapies. However, there are several significant gaps in our molecular and clinical understanding, as well as experimental challenges as discussed earlier. These gaps or hurdles must first be overcome before we can harness the clinical benefits of Klotho-based therapies as an elixir for vascular disease treatment or prevention. However, it seems likely that the pleiotropic nature of Klotho has brought together investigators from multiple different basic science and clinical disciplines that would have otherwise had traditionally disparate research emphases to work together toward a concerted strategy. What is interesting about a potential Klotho-based therapy is the possibility for a single drug to have multiple different disease indications. Transcending traditional barriers between disciplines offers immense opportunities for speeding innovative research that can address the growing burden of non-communicable diseases, in this case age-associated vascular diseases that remain a significant public health burden today.

## Figures and Tables

**Table 1:** Arterial Klotho expression in human and animal aortas. CKD, chronic kidney disease; VDRA, vitamin D receptor agonist.

Arterial Klotho Expression	Experimental Observations	Reference
Decreased mRNA and protein in human CKD	Human aorta Klotho deficiency in CKD can be reversed and calcification is attenuated ex vivo with VDRA	Lim et al. [13]
Decreased mRNA and protein in CKD mice	Low aortic Klotho but high circulating Klotho associated with vascular calcification in ldlr ^-/-^ CKD mice	Fang et al. [78]
mRNA but no protein in mouse aorta	Aortic Klotho has no role in vascular calcification	Lindberg et al. [79]
mRNA in human aorta, coronary arteries and thrombus	Klotho mRNA detectable in human arteries and thrombi of occlusive coronary disease	Donate-Correa et al. [81]
Increased mRNA and protein in calcified aorta of Enpp1^-/-^ mice	Increased Klotho associated with decreased vascular calcification in CKD mice	Zhu et al. [82]
mRNA and protein expression in rat aorta but not in rat vascular smooth muscle cells	No native VSMC Klotho expression, however overexpression worsens calcification	Jimbo et al. [50]
No mRNA or protein expression in mouse aorta	VDRA in vivo increases plasma αKlotho an decreases vascular calcification in CKD mice	Lau et al. [69]
No mRNA in normal and calcified aortas of CKD mice	No aortic Klotho expression and no Klotho effect in vitro	Scialla et al. [83]
